# Supplementary material for: The AaLplus near-peer teaching program in Family Medicine strengthens basic medical skills—A five-year retrospective study
Source: PLoS One. 2020 May 29;15(5):e0233748. doi: 10.1371/journal.pone.0233748 (PMC7259978; doi:10.1371/journal.pone.0233748)
Supplement: Attachment — Submitted filename: Response to Reviewers.docx [file pone.0233748.s003.docx]

Dear reviewers,

thank you very much for your extensive review which was very helpful for us to improve the manuscript. We now answer to each of your remarks.

Title: I think it is “a retrospective study” not cross-sectional, you could state “an analysis of five-year experience.”

We agree, thank you. We found a wording that indicates the retrospective nature of the study but does not suggest that our experience with the AaLplus program is limited to five years (which it is not)
Abstract: State what triggered the study. State the study research questions, not just aims.

Thank you for your remark. We rephrased the section and now give substantially more detail about research questions plus hypotheses.

Abstract - method: Provide more information such as how long did the participants use AaL plus program in each of the four semesters? What was the OSCE about? Did it cover history taking, clinical examination and procedures? How many stations? Not clear how feedback was given? Was feedback given by students (tutees) or also by standardized patients? Time of distribution of questionnaires? Was it before or after the OSCE?

We included concise information concerning the first four of your questions into the abstract. As to the latter four questions, we instead expanded on them in the methods section. Thank you for your detailed remarks.

Abstract- results: State number of females (% of females), state the SD, not just the MEAN, state the confidence interval. Were there differences between the results in the first year when you started the program and the results over the next years? How about maintenance issues, did you compare results in 2016, 2017 vs 2019, what was the trend, p values.

We now state the number of females and the SD in the abstract and briefly address the constant evaluation and further development. As to confidence intervals: All CIs for ordinal data (=students‘ self-rating scores) are either [2, 5] or [3, 5]. Due to lack of informational value to the reader, we suggest to omit these statistics. However, we now report confidence intervals for OSCE scores.

The feedback needs to clearly explained, the way it is written shows that there was no standardization or organization. Please give a clear description in the manuscript (not necessary in the abstract). In the abstract, the sentence needs to be rewritten. Words like “very” should not be used.

We now explain the feedback given to participants in more detail in the respective section below. We use quotation marks to indicate that „very helpful“ refers to the likert scale level.

Abstract- conclusion should be strengthened.

We included a sentence on the program’s routine viability, better reflecting our research hypotheses.

Background- State what triggered the study, State the research questions. Also, a third objective is to show that the program was maintained across these five years.

Thank you for this remark. We now state our research questions in detail.

We need to see if there were differences between when the course started compared to next years, and how the course was maintained and were there differences between the years 2016, 2017 and 2019.

We believe such inferencial comparisons do not aid substantially to answer our research questions. First, we are interested in providing a long-term view on the program’s effects and less on differences between the respective cohorts. Seconds, our program is constantly reevaluated and improved. Significant differences between cohorts‘ ratings or OSCE scores give us little additional insight, as we cannot validly attribute them to either program improvements, tutors‘ practice effect, changes/improvements in rating scales/rating behavior, cohort history effects, etc. Third, with a sample size of n>1500, inferential comparisons are trivial, because even slightest mean differences are statistically significant. Therefore, we report intraclass correlations for students‘ self-ratings. The low ICC statistics indicate that no further group comparison are helpful. In the discussion section, we now also briefly address this.

Methods- What do you mean by “all students (tutees) participated in the OSCE required…” This sentence needs to be clearly explained, It is not how “near peer-teaching” was organized, prepared, and how students were trained for this job.

We rephrased the sentence.

I see “AaL plus” repeated many times, giving an impression like a computer-aided program? Explain what do you mean by “AaL plus” very early in the manuscript.

Thank you for your remark. We rephrased many sentences, especially in the section „Skill acquisition through AaL*^plus^*“. We did not use a computer-aided program.

Describe the program and how it was developed.
Line 19, “In total, AaL plus encompasses 30 hours within two years, with every….” Again, not clear and should be clearly written, how long each session, how many sessions per week, what are the total hours per semester, how many hours in the two preclinical years. How these sessions matched with the blocks/modes in the curriculum?

We now give much more information on these details.

How were the standardized patients supervised? Did you train them? Details needed.

We now added information about supervision and references about the SP program we cooperate with.

Methods- The method section is crowded and not well organized. We have several issues AaL plus program, standardized patients, students, tutees (near-peer teaching), clinical exam, history, procedures, and others. You need to add two coloured flow diagrams explaining issues, (see for example the figures in the paper by Azer SA, et al. BMC Med Educ. 2013 May 24;13:71. doi: 10.1186/1472-6920-13-71). This part needs organization as well.

We reorganized the section, unified the use of terms, and added a synoptic flow diagram to explain the details of AaL*^plus^* in a convenient way.

Omit “cross-sectional”; it is a retrospective study. Briefly show how this program was integrated with other programs in the preclinical years, and then with Family Medicine later in the course.

AaLplus is aligned with other curricular blocks, including Medical Psychology lectures, Introduction to Careers in Medicine, and two mandatory one-day visitations in general practice. As proposed, we have added a few sentences on how the program is embedded into the whole curriculum.

What statistical analysis was used?
We need to see if there were differences between when the course started, how the course was maintained and were there differences between the years 2016, 2017 and 2019.

We used SPSS® for Windows, as stated in the „Statistical analysis“ subsection. Again, we suggest to not use inferential analysis.

Discussion and conclusions could be strengthened, in light of suggested changes.
We reworked the relevant details of the discussion and conclusion sections.

References: Some references are too old. Ref 28 covers the same idea here? Explain how this paper adds to what was studied earlier.

Ref 28 is a detailed (yet slightly outdated) program report. In the references, we now provide a translation for its title: [AaL*^plus^* — a preclinical course in history taking and physical examination techniques]

Reviewer #2: What is the role of the medical staff supervisors?

We now explain this in more detail: „These faculty staff members also join in lessons and OSCEs on a regular basis for the purpose of supervising tutors and standardized patients.“

In OSCE, the cases are real or dummies? With clinical finding or without? Who prepares and selects these cases for the exam?

Cases are not real, we use standardized patients. Dummies are only used for venepuncture practice. We added much more details to the standardized patients section, which should now answer your questions: „Lessons are supported by “standardized patients”, trained (amateur)semiprofessional actors who take on patient roles specifically designed for educational purposes. Standardized patients present symptoms and biographical information in a standardized way; they are selected, trained and supervised within the MediKIT program (Medizinisches Kommunikations- und Interaktionstraining [Medical Communication and Interaction Training])“

Lines 19-20 from page 11, what is the evidence for this conclusion?

We now give reference to these statements.

Lines 22-23 from page 11, what is the evidence for this conclusion?

This phrase is rather a hypothesis than e conclusion. We slightly changed the wording to make our line of thoughts clearer.

Line 2, page 12, is there evidence that these basic skills are especially fundamental for family medicine rather than other clinical specialties? The same comment applies to lines 6-7 page 13.

We rephrased and now refer more clearly to Franco et al., 2018

How can you explain the discrepancy between low scores in self-assessment for hand disinfection and blood pressure measurements on one hand and the relatively high scores in OSCE on the other hand?

We apologize, this must be a misunderstanding, the scores in self-assessment for these two skills are amongst the highest. Please let us know if we can rephrase a section to avoid this misunderstanding.
